# Bromide of Potassium in Epilepsy

**Published:** 1867-08

**Authors:** Horace Y. Evans


### Bromide of Potassium in Epilepsy.

BY HORACE Y. EVANS, M. D.

Dr. Evans relates the three following cases of this disease out of eight within his knowledge, treated with the bromides:

Case 1.—Farmer, aged thirty, living in a miasmatic region. Enjoyed perfect health until attacked with ague; was treated with quinia, and the chills checked. Then followed convulsions, which at first resembled, as far as the pulse was concerned, apoplexy, but soon became clearly epileptic. The attacks returned at irregular intervals of from seven to ten days. He had been carefully treated with remedies such as the symptoms from time to time indicated. When he came under my care he was using tonics and alteratives, and ice-bag to the spine. His pulse was 98, full and strong, tongue furred, bowels sluggish, disgust for food, very restless, severe headache, and marked mental confusion. I continued the ice-bag to his spine half an hour daily, ordered saline purge every day, and farinaceous diet. He was very soon visited by another convulsion, which left him in a dull melancholy condition, severe headache and insomnia, but no paralysis; commenced next day with the bromide of potassium, gr. xv, three times a day; continued the saline mixture, ice-bag, and restricted diet. An improvement in all the symptoms commenced within twelve hours, and at the expiration of four weeks the patient was apparently well; there was no return, or tendency to return, of the convulsion. All treatment was then omitted, and at the expiration of seven weeks from the commencement of the treatment, considering himself well, he returned to the use of animal food, which was followed within ten hours by the most severe epileptic fit of any that he had had, and two days later by another. He then returned to the city, and was again put upon the use of the bromide and the ice-bag. As at first, the improvement was rapid, and at the expiration of a fortnight, without my consent, omitted all treatment. He returned to the country, used promiscuous diet, and has now passed through the fever season of the locality without ague or convulsions. Says he was never in better health than at present.

Case 2.—G. M., a young man twenty-one years of age, apparently in good physical condition, has had epileptic convulsions for the past fifteen years, and at the time of commencing his treat ment (March, 1866,) he was having, on an average, three attacks a day. He was ordered a saline purge twice a week, ice-bag to spine one hour daily; bromide of potassium, gr. xx, three times a day, and total abstinence from animal food. The interruption in the attacks was immediate; he continued without even an "aura," or any other evidence of the presence of the disease for nine consecutive weeks.

The peculiar effects of the bromine, named by Bazire bromism, having now become developed, the drug was omitted for two days, Huxham's tincture of bark and a more liberal diet substituted. Before the end of the second day a severe convulsion returned, and was followed by numerous aura epileptica, or minor "spells." The bromide was immediately resumed, and its use continued for three weeks without a return of the disease. The increased flow of saliva, sore throat and restlessness again gave premonitions of the return of bromism. The dose was now reduced to gr. x, ter die. Again the lurking foe took advantage of the truce and made several sorties, which were repulsed by the bromide of ammonium, with the iodide of potassium as an ally. Another month now elapsed without an attack, but the combination last used became so offensive to him that it had to be omitted, and the bromide of potassium resumed in gr. xx doses, which is now (November) being used with results beyond the most sanguine anticipations.

Case 3.—Mrs. S. B., aged twenty-eight, the mother of two children. Insanity and epilepsy in her family. After a serious family trouble, was attacked with convulsions at intervals of a fortnight. The disease was diagnosed hysterical epilepsy, chiefly on account of the long duration of the convulsion. The usual treatment for hysteria scarcely palliated the insomnia and almost delirium during the intervals. Having seen an account of Locock's treatment of this disease with the bromide of potassium, I was induced to give it a trial. She commenced with gr. xx doses, three times a day, and an additional dose at night, if necessary, to produce sleep. Within a week every vestige of the disease had vanished. The medicine was continued in reduced doses for a month, after which it was entirely omitted. Four months have since passed without a symptom of hysteria or epilepsy, notwithstanding the continuance and actual increase of her family troubles.—*American Journal of Medical Sciences, January*, 1867.

